# Supplementary material for: Nitisinone Treatment Affects Biomarkers of Bone and Cartilage Remodelling in Alkaptonuria Patients
Source: Int J Mol Sci. 2023 Jul 1;24(13):10996. doi: 10.3390/ijms241310996 (PMC10341572; doi:10.3390/ijms241310996)

Table A1: Effect of nitisinone treatment on serum CTX-I after stratification for age, sex, cAKUSSI at baseline and concomitant treatment with antiresorptive treatment. The ratio of geometric means indicates the difference between nitisinone treatment and no treatment within strata. The ratio of ratios indicates the difference between the treatment effect across strata.

Visit	Ratio of ratios		
**Age**	**≤55 years**	**>55 years**	**Ratio**
**Year 1**	1.25 [1.07, 1.46]	1.09 [0.87, 1.37]	0.88 [0.67, 1.16]
**Year 2**	1.05 [0.90, 1.23]	0.95 [0.76, 1.19]	0.91 [0.69, 1.20]
**Year 3**	1.04 [0.88, 1.22]	0.82 [0.66, 1.03]	0.79 [0.60, 1.04]
**Year 4**	1.00 [0.85, 1.18]	0.91 [0.71, 1.15]	0.90 [0.68, 1.21]
**Sex**	**Male**	**Female**	
**Year 1**	1.13 [0.96, 1.33]	1.30 [1.05, 1.62]	1.15 [0.88, 1.51]
**Year 2**	1.02 [0.87, 1.20]	0.95 [0.77, 1.18]	0.93 [0.71, 1.22]
**Year 3**	0.97 [0.82, 1.14]	0.98 [0.79, 1.22]	1.02 [0.77, 1.34]
**Year 4**	0.93 [0.78, 1.10]	1.03 [0.82, 1.29]	1.11 [0.84, 1.47]
**cAKUSSI**	**Below median**	**Above median**	
**Year 1**	1.34 [1.11, 1.60]	1.07 [0.89, 1.28]	0.80 [0.62, 1.03]
**Year 2**	1.07 [0.90, 1.29]	0.93 [0.77, 1.11]	0.86 [0.67, 1.12]
**Year 3**	1.07 [0.89, 1.28]	0.88 [0.73, 1.06]	0.83 [0.64, 1.07]
**Year 4**	0.97 [0.80, 1.17]	0.95 [0.78, 1.15]	0.98 [0.75, 1.28]
**Antiresorptive treatment**	**Yes**	**No**	
**Year 1**	1.26 [1.06, 1.49]	1.11 [0.92, 1.33]	0.88 [0.68, 1.13]
**Year 2**	1.08 [0.91, 1.28]	0.89 [0.74, 1.08]	0.83 [0.64, 1.06]
**Year 3**	0.98 [0.82, 1.16]	0.95 [0.79, 1.15]	0.97 [0.75, 1.26]
**Year 4**	1.07 [0.89, 1.28]	0.84 [0.69, 1.02]	0.79 [0.60, 1.02]

Table A2: Effect of nitisinone treatment on serum PRO-C1 after stratification for age, sex, cAKUSSI at baseline and concomitant treatment with antiresorptive treatment. The ratio of geometric means indicates the difference between nitisinone treatment and no treatment within strata. The ratio of ratios indicates the difference between the treatment effect across strata.

**Visit**	**Ratio of ratios**		
**Age**	**≤55 years**	**>55 years**	**Ratio**
**Year 1**	1.46 [1.20, 1.78]	1.25 [0.94, 1.68]	0.86 [0.60, 1.22]
**Year 2**	1.21 [0.99, 1.48]	0.88 [0.66, 1.17]	0.73 [0.51, 1.03]
**Year 3**	1.04 [0.85, 1.28]	0.85 [0.64, 1.13]	0.82 [0.58, 1.16]
**Year 4**	0.94 [0.77, 1.16]	0.81 [0.60, 1.09]	0.85 [0.59, 1.23]
**Sex**	**Male**	**Female**	
**Year 1**	1.39 [1.13, 1.70]	1.39 [1.06, 1.84]	1.00 [0.71, 1.42]
**Year 2**	1.17 [0.95, 1.44]	0.93 [0.71, 1.23]	0.80 [0.56, 1.12]
**Year 3**	0.94 [0.77, 1.16]	1.01 [0.76, 1.33]	1.07 [0.76, 1.51]
**Year 4**	0.96 [0.78, 1.19]	0.80 [0.60, 1.07]	0.83 [0.58, 1.19]
**cAKUSSI**	**Below median**	**Above median**	
**Year 1**	1.48 [1.17, 1.87]	1.32 [1.04, 1.67]	0.89 [0.64, 1.24]
**Year 2**	1.15 [0.92, 1.45]	1.02 [0.80, 1.29]	0.88 [0.63, 1.22]
**Year 3**	0.99 [0.79, 1.25]	0.94 [0.74, 1.19]	0.95 [0.68, 1.32]
**Year 4**	0.85 [0.67, 1.08]	0.93 [0.73, 1.18]	1.09 [0.78, 1.54]
**Antiresorptive treatment**	**Yes**	**No**	
**Year 1**	1.39 [1.11, 1.73]	1.40 [1.10, 1.78]	1.01 [0.73, 1.40]
**Year 2**	1.19 [0.95, 1.48]	0.96 [0.75, 1.22]	0.81 [0.58, 1.12]
**Year 3**	1.08 [0.86, 1.35]	0.84 [0.66, 1.07]	0.78 [0.56, 1.08]
**Year 4**	0.93 [0.74, 1.17]	0.83 [0.64, 1.07]	0.89 [0.64, 1.26]

Table A3: Effect of nitisinone treatment on urine CTX-II/Creat after stratification for age, sex, cAKUSSI at baseline and concomitant treatment with antiresorptive treatment. The ratio of geometric means indicates the difference between nitisinone treatment and no treatment within strata. The ratio of ratios indicates the difference between the treatment effect across strata.

Visit	Ratio of ratios		
**Age**	**≤55 years**	**>55 years**	**Ratio**
**Year 1**	0.79 [0.62, 0.99]	1.28 [0.91, 1.81]	1.63 [1.08, 2.47]
**Year 2**	0.94 [0.74, 1.19]	0.93 [0.65, 1.32]	0.99 [0.65, 1.51]
**Year 3**	0.60 [0.47, 0.76]	1.13 [0.80, 1.59]	1.89 [1.24, 2.88]
**Year 4**	0.56 [0.43, 0.73]	1.30 [0.89, 1.91]	2.31 [1.45, 3.67]
**Sex**	**Male**	**Female**	
**Year 1**	0.85 [0.67, 1.08]	1.04 [0.75, 1.44]	1.22 [0.81, 1.84]
**Year 2**	0.94 [0.74, 1.20]	0.94 [0.67, 1.30]	0.99 [0.66, 1.50]
**Year 3**	0.67 [0.52, 0.86]	0.81 [0.58, 1.14]	1.22 [0.80, 1.85]
**Year 4**	0.59 [0.45, 0.77]	1.15 [0.80, 1.65]	1.95 [1.24, 3.07]
**cAKUSSI**	**Below median**	**Above median**	
**Year 1**	0.86 [0.65, 1.13]	1.00 [0.76, 1.33]	1.17 [0.79, 1.74]
**Year 2**	1.07 [0.81, 1.41]	0.83 [0.62, 1.10]	0.77 [0.52, 1.15]
**Year 3**	0.70 [0.53, 0.93]	0.76 [0.57, 1.02]	1.09 [0.73, 1.62]
**Year 4**	0.59 [0.44, 0.81]	0.92 [0.67, 1.26]	1.56 [1.00, 2.42]
**Antiresorptive treatment**	**Yes**	**No**	
**Year 1**	0.91 [0.70, 1.19]	0.94 [0.70, 1.26]	1.03 [0.70, 1.53]
**Year 2**	1.12 [0.86, 1.45]	0.77 [0.57, 1.03]	0.69 [0.46, 1.02]
**Year 3**	0.82 [0.63, 1.08]	0.62 [0.46, 0.83]	0.76 [0.51, 1.13]
**Year 4**	0.71 [0.53, 0.95]	0.76 [0.55, 1.04]	1.07 [0.69, 1.66]

Table A4: Effect of nitisinone treatment on serum C2M after stratification for age, sex, cAKUSSI at baseline and concomitant treatment with antiresorptive treatment. The ratio of geometric means indicates the difference between nitisinone treatment and no treatment within strata. The ratio of ratios indicates the difference between the treatment effect across strata.

Visit	Ratio of ratios		
**Age**	**≤55 years**	**>55 years**	**Ratio**
**Year 1**	0.89 [0.82, 0.97]	0.93 [0.82, 1.04]	1.04 [0.89, 1.20]
**Year 2**	0.87 [0.80, 0.95]	0.97 [0.86, 1.09]	1.11 [0.96, 1.28]
**Year 3**	0.90 [0.82, 0.98]	0.97 [0.86, 1.09]	1.08 [0.93, 1.25]
**Year 4**	0.87 [0.80, 0.95]	1.02 [0.90, 1.16]	1.17 [1.01, 1.37]
**Sex**	**Male**	**Female**	
**Year 1**	0.89 [0.82, 0.97]	0.93 [0.83, 1.04]	1.04 [0.90, 1.20]
**Year 2**	0.94 [0.86, 1.02]	0.87 [0.77, 0.97]	0.93 [0.80, 1.07]
**Year 3**	0.91 [0.83, 0.99]	0.95 [0.85, 1.07]	1.05 [0.91, 1.21]
**Year 4**	0.92 [0.84, 1.01]	0.90 [0.80, 1.02]	0.98 [0.84, 1.14]
**cAKUSSI**	**Below median**	**Above median**	
**Year 1**	0.87 [0.79, 0.96]	0.94 [0.86, 1.04]	1.08 [0.95, 1.24]
**Year 2**	0.87 [0.79, 0.96]	0.94 [0.86, 1.04]	1.08 [0.95, 1.24]
**Year 3**	0.90 [0.81, 0.99]	0.95 [0.86, 1.05]	1.06 [0.92, 1.21]
**Year 4**	0.83 [0.75, 0.92]	0.99 [0.89, 1.09]	1.18 [1.02, 1.37]
**Antiresorptive treatment**	**Yes**	**No**	
**Year 1**	0.87 [0.79, 0.95]	0.95 [0.86, 1.05]	1.10 [0.96, 1.26]
**Year 2**	0.90 [0.83, 0.99]	0.90 [0.81, 1.00]	1.00 [0.87, 1.14]
**Year 3**	0.93 [0.85, 1.02]	0.91 [0.82, 1.00]	0.97 [0.85, 1.11]
**Year 4**	0.91 [0.82, 1.00]	0.90 [0.81, 1.00]	0.99 [0.86, 1.14]

Table A5: Effect of nitisinone treatment on serum ALP after stratification for age, sex, cAKUSSI at baseline and concomitant treatment with antiresorptive treatment. The ratio of geometric means indicates the difference between nitisinone treatment and no treatment within strata. The ratio of ratios indicates the difference between the treatment effect across strata.

**Visit**	**Ratio of ratios**		
**Age**	**≤55 years**	**>55 years**	**Ratio**
**Year 1**	1.24 [1.14, 1.35]	1.22 [1.08, 1.37]	0.98 [0.85, 1.13]
**Year 2**	1.15 [1.06, 1.25]	1.16 [1.02, 1.31]	1.00 [0.87, 1.17]
**Year 3**	1.14 [1.04, 1.24]	1.15 [1.01, 1.29]	1.01 [0.87, 1.17]
**Year 4**	1.11 [1.02, 1.21]	1.06 [0.93, 1.20]	0.95 [0.82, 1.11]
**Sex**	**Male**	**Female**	
**Year 1**	1.19 [1.09, 1.30]	1.29 [1.16, 1.44]	1.09 [0.94, 1.25]
**Year 2**	1.16 [1.07, 1.26]	1.15 [1.03, 1.29]	0.99 [0.86, 1.15]
**Year 3**	1.12 [1.02, 1.22]	1.20 [1.07, 1.35]	1.08 [0.93, 1.24]
**Year 4**	1.08 [0.99, 1.19]	1.14 [1.01, 1.28]	1.05 [0.91, 1.21]
**cAKUSSI**	**Below median**	**Above median**	
**Year 1**	1.23 [1.12, 1.35]	1.23 [1.12, 1.36]	1.00 [0.88, 1.15]
**Year 2**	1.11 [1.01, 1.22]	1.20 [1.09, 1.32]	1.08 [0.94, 1.24]
**Year 3**	1.12 [1.02, 1.24]	1.17 [1.06, 1.29]	1.04 [0.91, 1.20]
**Year 4**	1.06 [0.96, 1.17]	1.12 [1.02, 1.24]	1.06 [0.92, 1.23]
**Antiresorptive treatment**	**Yes**	**No**	
**Year 1**	1.24 [1.13, 1.36]	1.20 [1.09, 1.33]	0.97 [0.85, 1.11]
**Year 2**	1.14 [1.04, 1.25]	1.16 [1.05, 1.29]	1.02 [0.89, 1.17]
**Year 3**	1.17 [1.06, 1.29]	1.11 [1.00, 1.23]	0.95 [0.82, 1.09]
**Year 4**	1.08 [0.98, 1.19]	1.10 [0.99, 1.22]	1.02 [0.89, 1.18]

Table A6: Concentration of the biomarkers at baseline (median and 95% confidence interval) in patients treated with antiresorptive treatment (bisphosphonates, vitamin D, systemic glucocorticoids and calcium) and in untreated patients. CTX-I, PRO-C1 and C2M (ng/mL), and CTX-II normalized by levels of urine creatinine (CTX-II/Creat) (ng/mmol).

	Treated (N=61)	Not treated (N=77)	p value
**Serum CTX_I**			
Median (Q1, Q3)	0.5 (0.4, 0.6)	0.5 (0.4, 0.7)	0.407
**Serum PRO-C1**			
Median (Q1, Q3)	88.8 (55.2, 116.8)	100.3 (63.9, 128.9)	0.212
**Serum C2M**			
Median (Q1, Q3)	0.3 (0.3, 0.4)	0.3 (0.3, 0.4)	0.890
**Urine CTX-II/Creat**			
Median (Q1, Q3)	7.0 (4.7, 11.1)	5.8 (3.8, 10.4)	0.396

Table A7: Concentration of alkaline phosphatase (ALP, U/L) at baseline in AKU patients according to nitisinone treatment, sex, age, cAKUSSI and antiresorptive treatment.

		ALP (Median, Q1, Q3)	p value
**Treatment**	Nitisinone	76.0 (65.0, 96.0)	0.273
	No treatment	75.0 (60.0, 90.0)	
**Sex**	Male	77.5 (63.7, 91.2)	0.352
	Female	76.0 (58.0, 92.0)	
**Age**	≤55 years	70.0 (59.0, 84.0)	< 0.001
	>55 years	84.0 (74.2, 100.5)	
**cAKUSSI**	Below median	66.0 (55.5, 78.0)	< 0.001
	Above median	84.0 (68.0, 100.0)	
**Antiresorptive treatment**	Yes	78.0 (65.0, 96.0)	0.112
	No	73.5 (59.7, 84.5)	

Table A8. Determination of cAKUSSI

Clinical AKU Severity Score Index (cAKUSSI)					
Feature		Score	Feature		Score
CLINICAL FEATURES (excluding spine and joint)					
Eye pigment (Standardised Medical Photography)					
Right eye (Nasal)	Slight	4	Left eye (Nasal)	Slight	4
	Marked	8		Marked	8
Right eye (Temporal)	Slight	4	Left eye (Temporal)	Slight	4
	Marked	8		Marked	8
Ear pigment (Standardised Medical Photography)					
Right ear	Slight	2	Left ear	Slight	2
	Marked	4		Marked	4
Stones (Ultrasonography of abdomen and pelvis)					
Prostate Stones	Per episode	4	Renal Stones	Per episode	4
Musculoskeletal					
Bone mineral density of hip<br>Dual Energy X-Ray Absorbtiometry (DEXA)	Grade (T-scores)<br>≥ -1·0<br>-1·0 to -1·7<br>-1·8 to -2·4<br>< -2·5	0<br>2<br>4<br>6			
Adult fracture (Questionnaire)	Per fracture	8	Ligament rupture	Per rupture	8
Tendon rupture (Questionnaire)	Per rupture	8	Muscle rupture	Per rupture	8
Heart (Transthoracic echocardiography)					
Normal<br>Aortic sclerosis		0<br>4	Aortic valve stenosis	Mild<br>Moderate<br>Severe	8<br>10<br>12
ENT (Audiometry)					
Hearing impairment	Grade on audiometry (dB loss), per ear<br>≤ 20	0	Dark tympanic membrane (Otoscopic	Per ear	6

Clinical AKU Severity Score Index (cAKUSSI)					
Feature		Score	Feature		Score
	21-35 (mild)<br>36-60 (moderate)<br>>60 (severe)	1<br>2<br>4	examination)		
JOINT FEATURES					
Clinical joint pain (1 for each large joint area; hips, knees, ankles, feet, shoulders, elbows, wrists & hands - right and left sides = 14 joint areas) (Questionnaire)					Max 14
Non-spine joint disease (2 for each large joint area; hips, knees, ankles, feet, shoulders, elbows, wrists & hands - right and left sides = 14 joint areas) (either Technetium 99m-methyl diphosphonate or 18FPETCT)					Max 28
Arthroscopies (Questionnaire)					2 each
Joint replacements (Questionnaire)					4 each
SPINE FEATURES					
Clinical spinal pain (2 each for cervical, thoracic, lumbar, sacroiliac) (Questionnaire)					Max 8
Spine disease (4 each for pubic symphysis, ribs, sacroiliac, lumbar, thoracic, cervical) (either Technetium 99m-methyl diphosphonate or 18FPETCT)					Max 24
Kyphosis<br>(X-Ray Lateral Spine and pelvis)	(Cobb angles)<br><45<br>45.-60<br>>60	 0<br>3<br>6	Scoliosis<br>X-Ray antero-posterior Spine and pelvis	(Cobb angles)<br><5<br>5-20<br>21-30<br>>30	 0<br>2<br>4<br>6

Figure A1. Serum ALP concentrations over time in untreated controls and nitisinone-treated patients. Estimated geometric means with 95% confidence intervals.

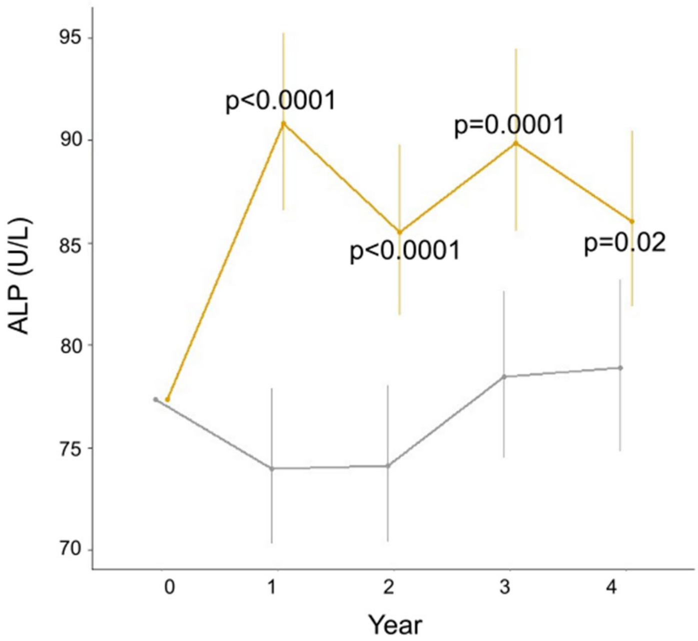

Figure A2. Flow chart of SONIA 2 biomarker study

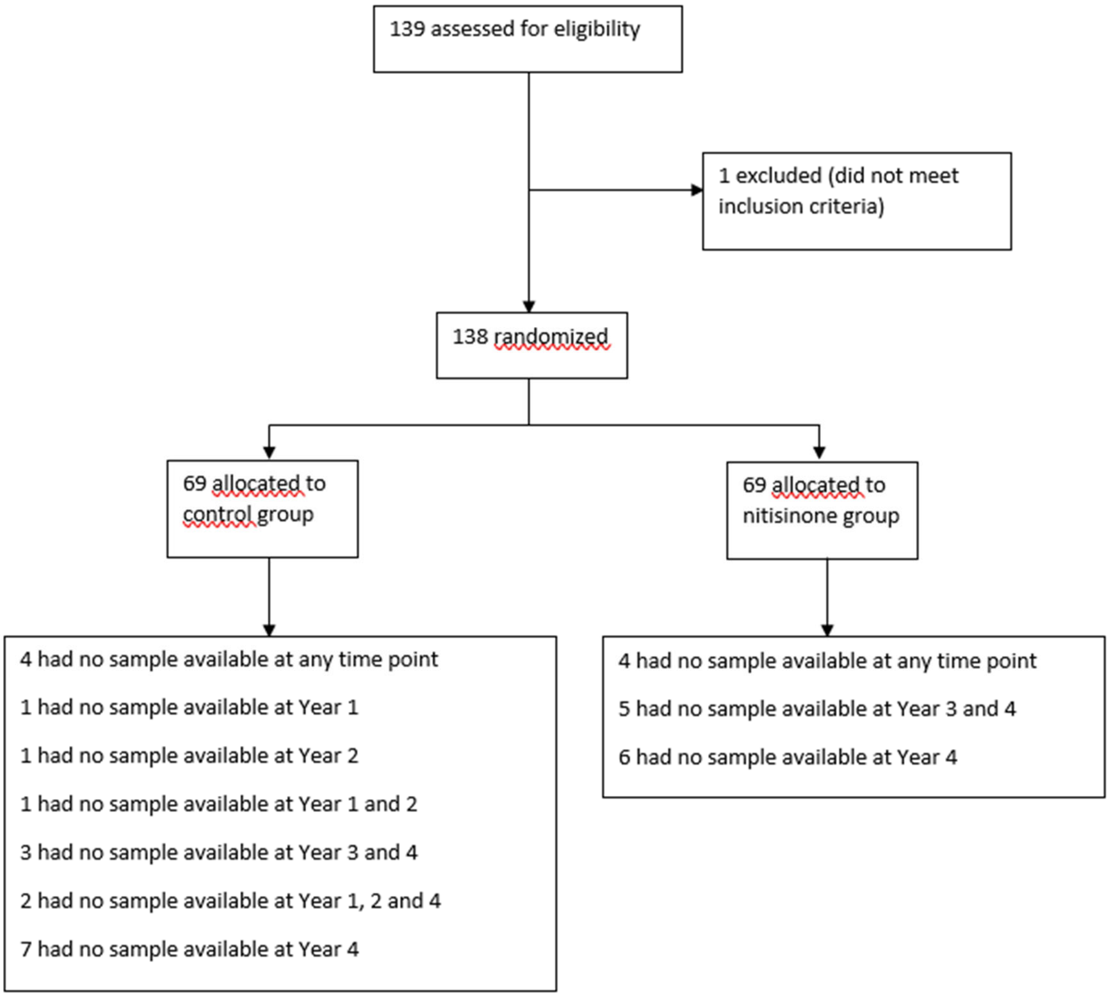

Supplement: Supplementary file 1 [file ijms-24-10996-s001.zip › ijms-2429237-supplementary.pdf]
